# Supplementary material for: Nested Melanoma and Superficial Spreading Melanoma with Prominent Nests—A Retrospective Study on Clinical Characteristics and PRAME Expression
Source: Diagnostics (Basel). 2025 Sep 8;15(17):2279. doi: 10.3390/diagnostics15172279 (PMC12428223; doi:10.3390/diagnostics15172279)

Supplementary Figure S1: Flow chart of case selection with inclusion and exclusion criteria.

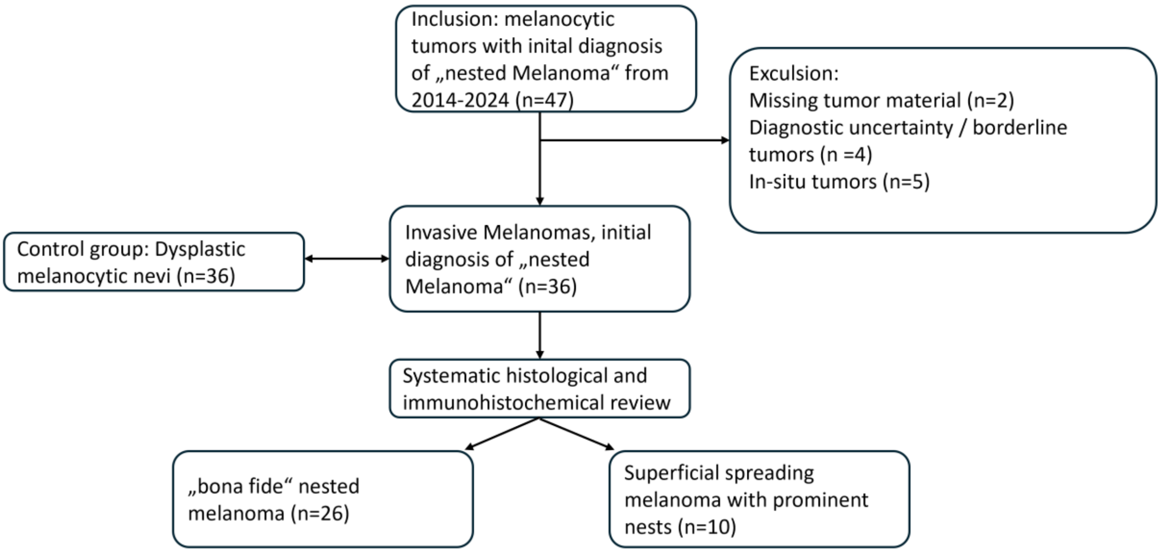

Supplementary Figure S2: (a,b) (patient no. 48): dysplastic melanocytic compound nevus with bridging of melanocytes and dermal nevoid melanocytes with maturation towards deeper layers (a). PRAME is positively expressed in individual epidermal and dermal melanocytes (arrows), but diffuse expression pattern is not found. Scale bar is 100 µm.

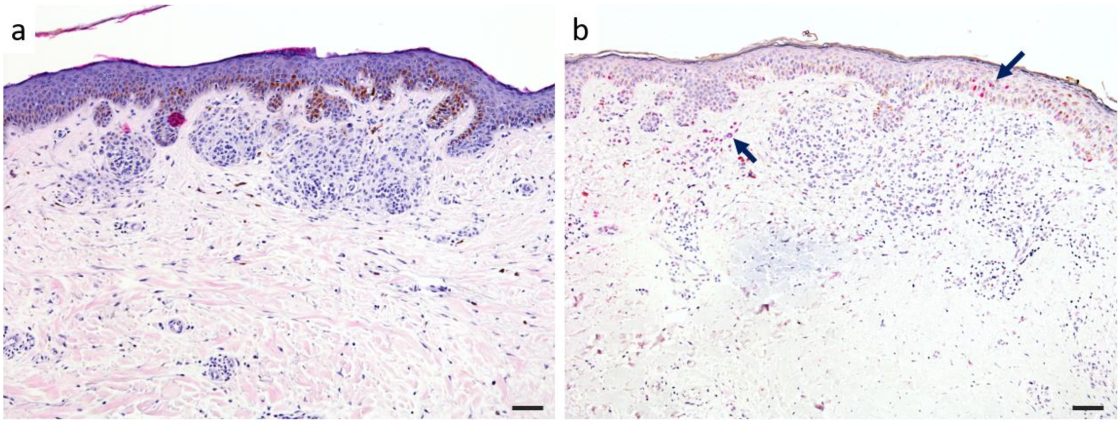

Supplement: Supplementary file 1 [file diagnostics-15-02279-s001.zip › diagnostics-3835124-supplementary.pdf]
